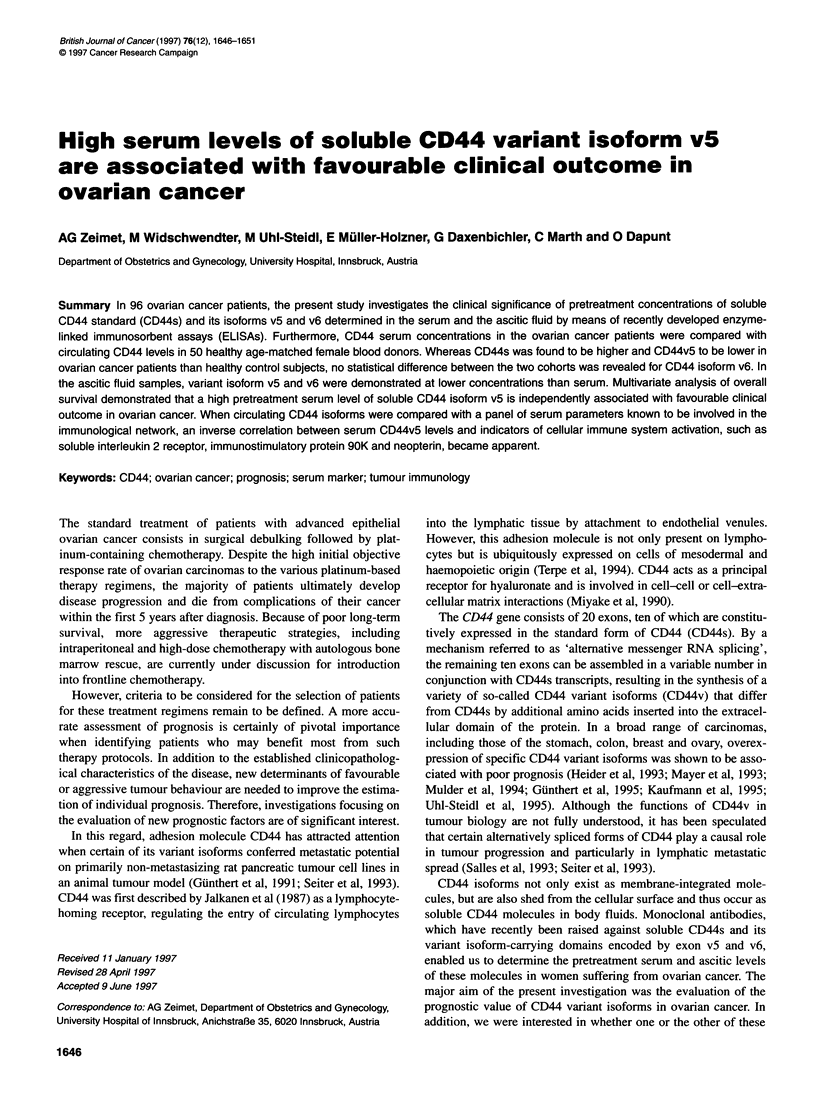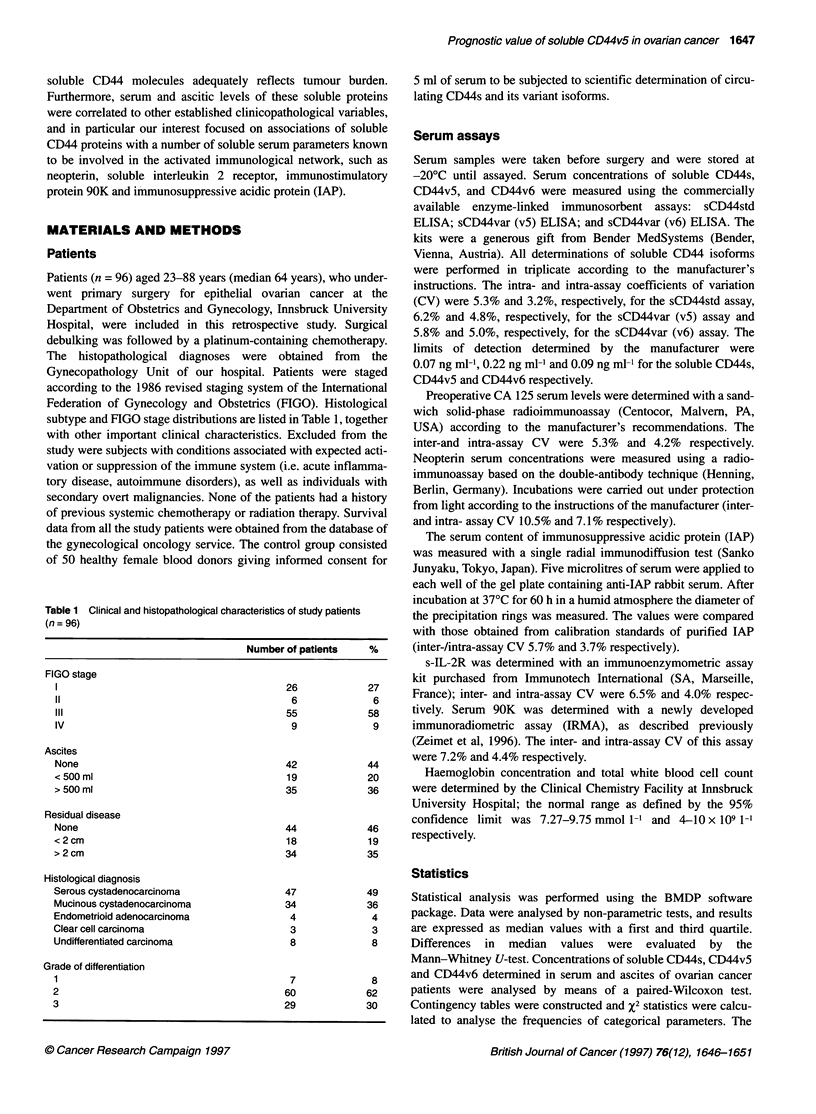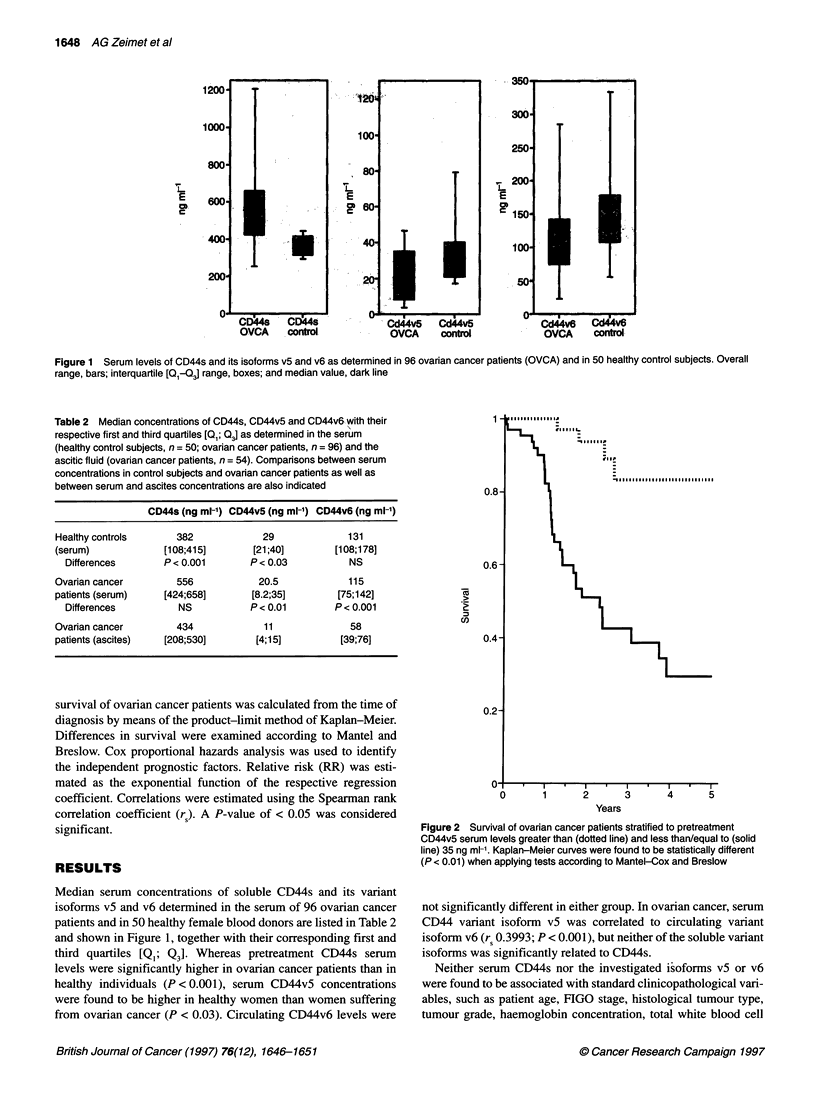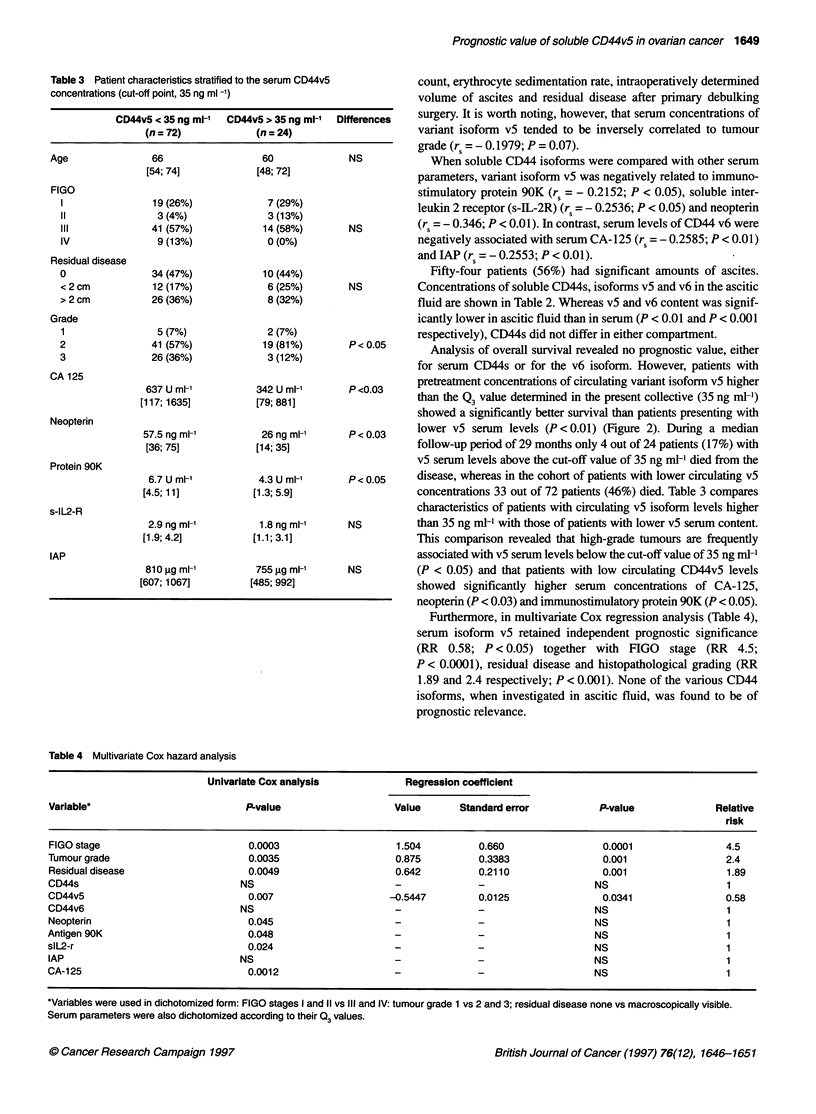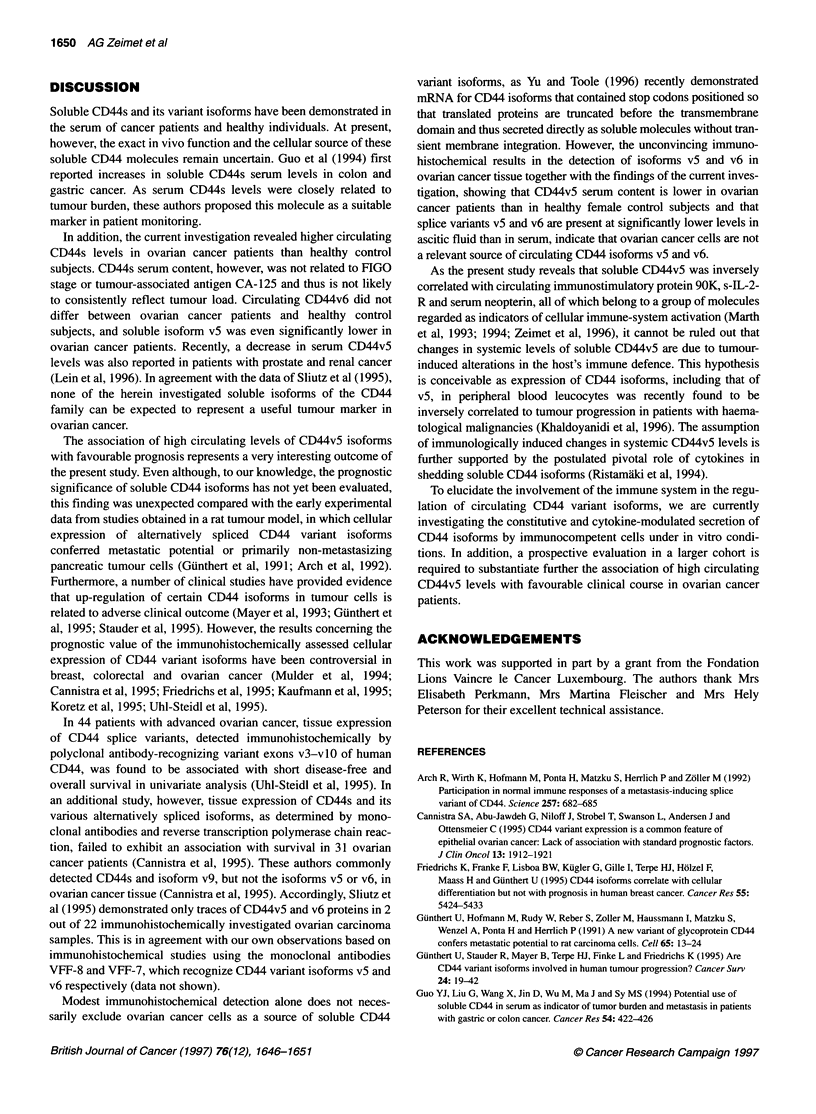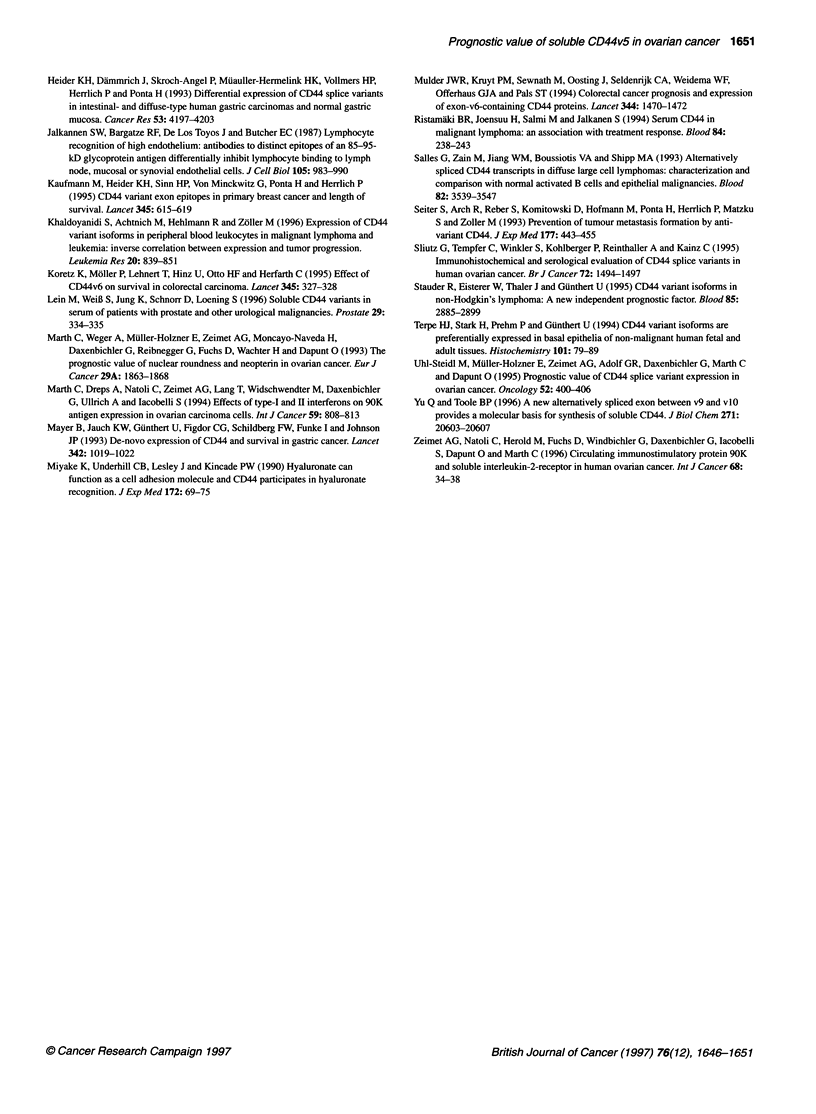# High serum levels of soluble CD44 variant isoform v5 are associated with favourable clinical outcome in ovarian cancer.

**DOI:** 10.1038/bjc.1997.611

**Published:** 1997

**Authors:** A. G. Zeimet, M. Widschwendter, M. Uhl-Steidl, E. MÃ¼ller-Holzner, G. Daxenbichler, C. Marth, O. Dapunt

**Affiliations:** Department of Obstetrics and Gynecology, University Hospital, Innsbruck, Austria.

## Abstract

In 96 ovarian cancer patients, the present study investigates the clinical significance of pretreatment concentrations of soluble CD44 standard (CD44s) and its isoforms v5 and v6 determined in the serum and the ascitic fluid by means of recently developed enzyme-linked immunosorbent assays (ELISAs). Furthermore, CD44 serum concentrations in the ovarian cancer patients were compared with circulating CD44 levels in 50 healthy age-matched female blood donors. Whereas CD44s was found to be higher and CD44v5 to be lower in ovarian cancer patients than healthy control subjects, no statistical difference between the two cohorts was revealed for CD44 isoform v6. In the ascitic fluid samples, variant isoform v5 and v6 were demonstrated at lower concentrations than serum. Multivariate analysis of overall survival demonstrated that a high pretreatment serum level of soluble CD44 isoform v5 is independently associated with favourable clinical outcome in ovarian cancer. When circulating CD44 isoforms were compared with a panel of serum parameters known to be involved in the immunological network, an inverse correlation between serum CD44v5 levels and indicators of cellular immune system activation, such as soluble interleukin 2 receptor, immunostimulatory protein 90K and neopterin, became apparent.


British Journal of Cancer (1997) 76(12), 1646–1651
© 1997 Cancer Research Campaign

### High serum levels of soluble CD44 variant isoform v5 are associated with favourable clinical outcome in ovarian cancer

**AG Zeimet, M Widschwendter, M Uhl-Steidl, E Müller-Holzner, G Daxenbichler, C Marth and O Dapunt**

Department of Obstetrics and Gynecology, University Hospital, Innsbruck, Austria

**Summary** In 96 ovarian cancer patients, the present study investigates the clinical significance of pretreatment concentrations of soluble CD44 standard (CD44s) and its isoforms v5 and v6 determined in the serum and the ascitic fluid by means of recently developed enzyme-linked immunosorbent assays (ELISAs). Furthermore, CD44 serum concentrations in the ovarian cancer patients were compared with circulating CD44 levels in 50 healthy age-matched female blood donors. Whereas CD44s was found to be higher and CD44v5 to be lower in ovarian cancer patients than healthy control subjects, no statistical difference between the two cohorts was revealed for CD44 isoform v6. In the ascitic fluid samples, variant isoform v5 and v6 were demonstrated at lower concentrations than serum. Multivariate analysis of overall survival demonstrated that a high pretreatment serum level of soluble CD44 isoform v5 is independently associated with favourable clinical outcome in ovarian cancer. When circulating CD44 isoforms were compared with a panel of serum parameters known to be involved in the immunological network, an inverse correlation between serum CD44v5 levels and indicators of cellular immune system activation, such as soluble interleukin 2 receptor, immunostimulatory protein 90K and neopterin, became apparent.

**Keywords:** CD44; ovarian cancer; prognosis; serum marker; tumour immunology

The standard treatment of patients with advanced epithelial ovarian cancer consists in surgical debulking followed by platinum-containing chemotherapy. Despite the high initial objective response rate of ovarian carcinomas to the various platinum-based therapy regimens, the majority of patients ultimately develop disease progression and die from complications of their cancer within the first 5 years after diagnosis. Because of poor long-term survival, more aggressive therapeutic strategies, including intraperitoneal and high-dose chemotherapy with autologous bone marrow rescue, are currently under discussion for introduction into frontline chemotherapy.

However, criteria to be considered for the selection of patients for these treatment regimens remain to be defined. A more accurate assessment of prognosis is certainly of pivotal importance when identifying patients who may benefit most from such therapy protocols. In addition to the established clinicopathological characteristics of the disease, new determinants of favourable or aggressive tumour behaviour are needed to improve the estimation of individual prognosis. Therefore, investigations focusing on the evaluation of new prognostic factors are of significant interest.

In this regard, adhesion molecule CD44 has attracted attention when certain of its variant isoforms conferred metastatic potential on primarily non-metastasizing rat pancreatic tumour cell lines in an animal tumour model (Günthert et al, 1991; Seiter et al, 1993). CD44 was first described by Jalkanen et al (1987) as a lymphocyte-homing receptor, regulating the entry of circulating lymphocytes into the lymphatic tissue by attachment to endothelial venules. However, this adhesion molecule is not only present on lymphocytes but is ubiquitously expressed on cells of mesodermal and haemopoietic origin (Terpe et al, 1994). CD44 acts as a principal receptor for hyaluronate and is involved in cell–cell or cell–extracellular matrix interactions (Miyake et al, 1990).

The *CD44* gene consists of 20 exons, ten of which are constitutively expressed in the standard form of CD44 (CD44s). By a mechanism referred to as 'alternative messenger RNA splicing', the remaining ten exons can be assembled in a variable number in conjunction with CD44s transcripts, resulting in the synthesis of a variety of so-called CD44 variant isoforms (CD44v) that differ from CD44s by additional amino acids inserted into the extracellular domain of the protein. In a broad range of carcinomas, including those of the stomach, colon, breast and ovary, overexpression of specific CD44 variant isoforms was shown to be associated with poor prognosis (Heider et al, 1993; Mayer et al, 1993; Mulder et al, 1994; Günthert et al, 1995; Kaufmann et al, 1995; Uhl-Steidl et al, 1995). Although the functions of CD44v in tumour biology are not fully understood, it has been speculated that certain alternatively spliced forms of CD44 play a causal role in tumour progression and particularly in lymphatic metastatic spread (Salles et al, 1993; Seiter et al, 1993).

CD44 isoforms not only exist as membrane-integrated molecules, but are also shed from the cellular surface and thus occur as soluble CD44 molecules in body fluids. Monoclonal antibodies, which have recently been raised against soluble CD44s and its variant isoform-carrying domains encoded by exon v5 and v6, enabled us to determine the pretreatment serum and ascitic levels of these molecules in women suffering from ovarian cancer. The major aim of the present investigation was the evaluation of the prognostic value of CD44 variant isoforms in ovarian cancer. In addition, we were interested in whether one or the other of these

*Received 11 January 1997*
*Revised 28 April 1997*
*Accepted 9 June 1997*

*Correspondence to:* AG Zeimet, Department of Obstetrics and Gynecology, University Hospital of Innsbruck, Anichstraße 35, 6020 Innsbruck, Austria soluble CD44 molecules adequately reflects tumour burden. Furthermore, serum and ascitic levels of these soluble proteins were correlated to other established clinicopathological variables, and in particular our interest focused on associations of soluble CD44 proteins with a number of soluble serum parameters known to be involved in the activated immunological network, such as neopterin, soluble interleukin 2 receptor, immunostimulatory protein 90K and immunosuppressive acidic protein (IAP).

### MATERIALS AND METHODS

### Patients

Patients ($n = 96$) aged 23–88 years (median 64 years), who underwent primary surgery for epithelial ovarian cancer at the Department of Obstetrics and Gynecology, Innsbruck University Hospital, were included in this retrospective study. Surgical debulking was followed by a platinum-containing chemotherapy. The histopathological diagnoses were obtained from the Gynecopathology Unit of our hospital. Patients were staged according to the 1986 revised staging system of the International Federation of Gynecology and Obstetrics (FIGO). Histological subtype and FIGO stage distributions are listed in Table 1, together with other important clinical characteristics. Excluded from the study were subjects with conditions associated with expected activation or suppression of the immune system (i.e. acute inflammatory disease, autoimmune disorders), as well as individuals with secondary overt malignancies. None of the patients had a history of previous systemic chemotherapy or radiation therapy. Survival data from all the study patients were obtained from the database of the gynecological oncology service. The control group consisted of 50 healthy female blood donors giving informed consent for 5 ml of serum to be subjected to scientific determination of circulating CD44s and its variant isoforms.

**Table 1** Clinical and histopathological characteristics of study patients ($n = 96$)

| | Number of patients | % |
|---|---|---|
| FIGO stage | | |
| I | 26 | 27 |
| II | 6 | 6 |
| III | 55 | 58 |
| IV | 9 | 9 |
| Ascites | | |
| None | 42 | 44 |
| < 500 ml | 19 | 20 |
| > 500 ml | 35 | 36 |
| Residual disease | | |
| None | 44 | 46 |
| < 2 cm | 18 | 19 |
| > 2 cm | 34 | 35 |
| Histological diagnosis | | |
| Serous cystadenocarcinoma | 47 | 49 |
| Mucinous cystadenocarcinoma | 34 | 36 |
| Endometrioid adenocarcinoma | 4 | 4 |
| Clear cell carcinoma | 3 | 3 |
| Undifferentiated carcinoma | 8 | 8 |
| Grade of differentiation | | |
| 1 | 7 | 8 |
| 2 | 60 | 62 |
| 3 | 29 | 30 |

### Serum assays

Serum samples were taken before surgery and were stored at –20°C until assayed. Serum concentrations of soluble CD44s, CD44v5, and CD44v6 were measured using the commercially available enzyme-linked immunosorbent assays: sCD44std ELISA; sCD44var (v5) ELISA; and sCD44var (v6) ELISA. The kits were a generous gift from Bender MedSystems (Bender, Vienna, Austria). All determinations of soluble CD44 isoforms were performed in triplicate according to the manufacturer's instructions. The intra- and intra-assay coefficients of variation (CV) were 5.3% and 3.2%, respectively, for the sCD44std assay, 6.2% and 4.8%, respectively, for the sCD44var (v5) assay and 5.8% and 5.0%, respectively, for the sCD44var (v6) assay. The limits of detection determined by the manufacturer were 0.07 ng $ml^{-1}$, 0.22 ng $ml^{-1}$ and 0.09 ng $ml^{-1}$ for the soluble CD44s, CD44v5 and CD44v6 respectively.

Preoperative CA 125 serum levels were determined with a sandwich solid-phase radioimmunoassay (Centocor, Malvern, PA, USA) according to the manufacturer's recommendations. The inter-and intra-assay CV were 5.3% and 4.2% respectively. Neopterin serum concentrations were measured using a radioimmunoassay based on the double-antibody technique (Henning, Berlin, Germany). Incubations were carried out under protection from light according to the instructions of the manufacturer (inter- and intra- assay CV 10.5% and 7.1% respectively).

The serum content of immunosuppressive acidic protein (IAP) was measured with a single radial immunodiffusion test (Sanko Junyaku, Tokyo, Japan). Five microlitres of serum were applied to each well of the gel plate containing anti-IAP rabbit serum. After incubation at 37°C for 60 h in a humid atmosphere the diameter of the precipitation rings was measured. The values were compared with those obtained from calibration standards of purified IAP (inter-/intra-assay CV 5.7% and 3.7% respectively).

s-IL-2R was determined with an immunoenzymometric assay kit purchased from Immunotech International (SA, Marseille, France); inter- and intra-assay CV were 6.5% and 4.0% respectively. Serum 90K was determined with a newly developed immunoradiometric assay (IRMA), as described previously (Zeimet et al, 1996). The inter- and intra-assay CV of this assay were 7.2% and 4.4% respectively.

Haemoglobin concentration and total white blood cell count were determined by the Clinical Chemistry Facility at Innsbruck University Hospital; the normal range as defined by the 95% confidence limit was 7.27–9.75 mmol $l^{-1}$ and 4–10 × $10^9$ $l^{-1}$ respectively.

### Statistics

Statistical analysis was performed using the BMDP software package. Data were analysed by non-parametric tests, and results are expressed as median values with a first and third quartile. Differences in median values were evaluated by the Mann–Whitney $U$-test. Concentrations of soluble CD44s, CD44v5 and CD44v6 determined in serum and ascites of ovarian cancer patients were analysed by means of a paired-Wilcoxon test. Contingency tables were constructed and $\chi^2$ statistics were calculated to analyse the frequencies of categorical parameters. The

**Figure 1** Serum levels of CD44s and its isoforms v5 and v6 as determined in 96 ovarian cancer patients (OVCA) and in 50 healthy control subjects. Overall range, bars; interquartile [$Q_1$–$Q_3$] range, boxes; and median value, dark line

**Table 2** Median concentrations of CD44s, CD44v5 and CD44v6 with their respective first and third quartiles [$Q_1$; $Q_3$] as determined in the serum (healthy control subjects, $n = 50$; ovarian cancer patients, $n = 96$) and the ascitic fluid (ovarian cancer patients, $n = 54$). Comparisons between serum concentrations in control subjects and ovarian cancer patients as well as between serum and ascites concentrations are also indicated

| | CD44s (ng ml⁻¹) | CD44v5 (ng ml⁻¹) | CD44v6 (ng ml⁻¹) |
|---|---|---|---|
| Healthy controls (serum) | 382 [108;415] | 29 [21;40] | 131 [108;178] |
| Differences | $P < 0.001$ | $P < 0.03$ | NS |
| Ovarian cancer patients (serum) | 556 [424;658] | 20.5 [8.2;35] | 115 [75;142] |
| Differences | NS | $P < 0.01$ | $P < 0.001$ |
| Ovarian cancer patients (ascites) | 434 [208;530] | 11 [4;15] | 58 [39;76] |

survival of ovarian cancer patients was calculated from the time of diagnosis by means of the product–limit method of Kaplan–Meier. Differences in survival were examined according to Mantel and Breslow. Cox proportional hazards analysis was used to identify the independent prognostic factors. Relative risk (RR) was estimated as the exponential function of the respective regression coefficient. Correlations were estimated using the Spearman rank correlation coefficient ($r_s$). A $P$-value of < 0.05 was considered significant.

### RESULTS

Median serum concentrations of soluble CD44s and its variant isoforms v5 and v6 determined in the serum of 96 ovarian cancer patients and in 50 healthy female blood donors are listed in Table 2 and shown in Figure 1, together with their corresponding first and third quartiles [$Q_1$; $Q_3$]. Whereas pretreatment CD44s serum levels were significantly higher in ovarian cancer patients than in healthy individuals ($P < 0.001$), serum CD44v5 concentrations were found to be higher in healthy women than women suffering from ovarian cancer ($P < 0.03$). Circulating CD44v6 levels were not significantly different in either group. In ovarian cancer, serum CD44 variant isoform v5 was correlated to circulating variant isoform v6 ($r_s$ 0.3993; $P < 0.001$), but neither of the soluble variant isoforms was significantly related to CD44s.

**Figure 2** Survival of ovarian cancer patients stratified to pretreatment CD44v5 serum levels greater than (dotted line) and less than/equal to (solid line) 35 ng ml⁻¹. Kaplan–Meier curves were found to be statistically different ($P < 0.01$) when applying tests according to Mantel–Cox and Breslow

Neither serum CD44s nor the investigated isoforms v5 or v6 were found to be associated with standard clinicopathological variables, such as patient age, FIGO stage, histological tumour type, tumour grade, haemoglobin concentration, total white blood cell

© Cancer Research Campaign 1997

**Table 3** Patient characteristics stratified to the serum CD44v5 concentrations (cut-off point, 35 ng ml $^{-1}$)

| | CD44v5 < 35 ng ml$^{-1}$ (*n* = 72) | CD44v5 > 35 ng ml$^{-1}$ (*n* = 24) | Differences |
|---|---|---|---|
| Age | 66 [54; 74] | 60 [48; 72] | NS |
| FIGO | | | |
| I | 19 (26%) | 7 (29%) | |
| II | 3 (4%) | 3 (13%) | |
| III | 41 (57%) | 14 (58%) | NS |
| IV | 9 (13%) | 0 (0%) | |
| Residual disease | | | |
| 0 | 34 (47%) | 10 (44%) | |
| < 2 cm | 12 (17%) | 6 (25%) | NS |
| > 2 cm | 26 (36%) | 8 (32%) | |
| Grade | | | |
| 1 | 5 (7%) | 2 (7%) | |
| 2 | 41 (57%) | 19 (81%) | *P* < 0.05 |
| 3 | 26 (36%) | 3 (12%) | |
| CA 125 | 637 U ml$^{-1}$ [117; 1635] | 342 U ml$^{-1}$ [79; 881] | *P* <0.03 |
| Neopterin | 57.5 ng ml$^{-1}$ [36; 75] | 26 ng ml$^{-1}$ [14; 35] | *P* < 0.03 |
| Protein 90K | 6.7 U ml$^{-1}$ [4.5; 11] | 4.3 U ml$^{-1}$ [1.3; 5.9] | *P* < 0.05 |
| s-IL2-R | 2.9 ng ml$^{-1}$ [1.9; 4.2] | 1.8 ng ml$^{-1}$ [1.1; 3.1] | NS |
| IAP | 810 µg ml$^{-1}$ [607; 1067] | 755 µg ml$^{-1}$ [485; 992] | NS |

count, erythrocyte sedimentation rate, intraoperatively determined volume of ascites and residual disease after primary debulking surgery. It is worth noting, however, that serum concentrations of variant isoform v5 tended to be inversely correlated to tumour grade ($r_s$ = – 0.1979; $P$ = 0.07).

When soluble CD44 isoforms were compared with other serum parameters, variant isoform v5 was negatively related to immunostimulatory protein 90K ($r_s$ = – 0.2152; $P$ < 0.05), soluble interleukin 2 receptor (s-IL-2R) ($r_s$ = – 0.2536; $P$ < 0.05) and neopterin ($r_s$ = – 0.346; $P$ < 0.01). In contrast, serum levels of CD44 v6 were negatively associated with serum CA-125 ($r_s$ = – 0.2585; $P$ < 0.01) and IAP ($r_s$ = – 0.2553; $P$ < 0.01).

Fifty-four patients (56%) had significant amounts of ascites. Concentrations of soluble CD44s, isoforms v5 and v6 in the ascitic fluid are shown in Table 2. Whereas v5 and v6 content was significantly lower in ascitic fluid than in serum ($P$ < 0.01 and $P$ < 0.001 respectively), CD44s did not differ in either compartment.

Analysis of overall survival revealed no prognostic value, either for serum CD44s or for the v6 isoform. However, patients with pretreatment concentrations of circulating variant isoform v5 higher than the $Q_3$ value determined in the present collective (35 ng ml$^{-1}$) showed a significantly better survival than patients presenting with lower v5 serum levels ($P$ < 0.01) (Figure 2). During a median follow-up period of 29 months only 4 out of 24 patients (17%) with v5 serum levels above the cut-off value of 35 ng ml$^{-1}$ died from the disease, whereas in the cohort of patients with lower circulating v5 concentrations 33 out of 72 patients (46%) died. Table 3 compares characteristics of patients with circulating v5 isoform levels higher than 35 ng ml$^{-1}$ with those of patients with lower v5 serum content. This comparison revealed that high-grade tumours are frequently associated with v5 serum levels below the cut-off value of 35 ng ml$^{-1}$ ($P$ < 0.05) and that patients with low circulating CD44v5 levels showed significantly higher serum concentrations of CA-125, neopterin ($P$ < 0.03) and immunostimulatory protein 90K ($P$ < 0.05).

Furthermore, in multivariate Cox regression analysis (Table 4), serum isoform v5 retained independent prognostic significance (RR 0.58; $P$ < 0.05) together with FIGO stage (RR 4.5; $P$ < 0.0001), residual disease and histopathological grading (RR 1.89 and 2.4 respectively; $P$ < 0.001). None of the various CD44 isoforms, when investigated in ascitic fluid, was found to be of prognostic relevance.

**Table 4** Multivariate Cox hazard analysis

| Variable* | Univariate Cox analysis *P*-value | Regression coefficient Value | Regression coefficient Standard error | *P*-value | Relative risk |
|---|---|---|---|---|---|
| FIGO stage | 0.0003 | 1.504 | 0.660 | 0.0001 | 4.5 |
| Tumour grade | 0.0035 | 0.875 | 0.3383 | 0.001 | 2.4 |
| Residual disease | 0.0049 | 0.642 | 0.2110 | 0.001 | 1.89 |
| CD44s | NS | – | – | NS | 1 |
| CD44v5 | 0.007 | –0.5447 | 0.0125 | 0.0341 | 0.58 |
| CD44v6 | NS | – | – | NS | 1 |
| Neopterin | 0.045 | – | – | NS | 1 |
| Antigen 90K | 0.048 | – | – | NS | 1 |
| sIL2-r | 0.024 | – | – | NS | 1 |
| IAP | NS | – | – | NS | 1 |
| CA-125 | 0.0012 | – | – | NS | 1 |

*Variables were used in dichotomized form: FIGO stages I and II vs III and IV: tumour grade 1 vs 2 and 3; residual disease none vs macroscopically visible. Serum parameters were also dichotomized according to their $Q_3$ values.

### DISCUSSION

Soluble CD44s and its variant isoforms have been demonstrated in the serum of cancer patients and healthy individuals. At present, however, the exact in vivo function and the cellular source of these soluble CD44 molecules remain uncertain. Guo et al (1994) first reported increases in soluble CD44s serum levels in colon and gastric cancer. As serum CD44s levels were closely related to tumour burden, these authors proposed this molecule as a suitable marker in patient monitoring.

In addition, the current investigation revealed higher circulating CD44s levels in ovarian cancer patients than healthy control subjects. CD44s serum content, however, was not related to FIGO stage or tumour-associated antigen CA-125 and thus is not likely to consistently reflect tumour load. Circulating CD44v6 did not differ between ovarian cancer patients and healthy control subjects, and soluble isoform v5 was even significantly lower in ovarian cancer patients. Recently, a decrease in serum CD44v5 levels was also reported in patients with prostate and renal cancer (Lein et al, 1996). In agreement with the data of Sliutz et al (1995), none of the herein investigated soluble isoforms of the CD44 family can be expected to represent a useful tumour marker in ovarian cancer.

The association of high circulating levels of CD44v5 isoforms with favourable prognosis represents a very interesting outcome of the present study. Even although, to our knowledge, the prognostic significance of soluble CD44 isoforms has not yet been evaluated, this finding was unexpected compared with the early experimental data from studies obtained in a rat tumour model, in which cellular expression of alternatively spliced CD44 variant isoforms conferred metastatic potential or primarily non-metastasizing pancreatic tumour cells (Günthert et al, 1991; Arch et al, 1992). Furthermore, a number of clinical studies have provided evidence that up-regulation of certain CD44 isoforms in tumour cells is related to adverse clinical outcome (Mayer et al, 1993; Günthert et al, 1995; Stauder et al, 1995). However, the results concerning the prognostic value of the immunohistochemically assessed cellular expression of CD44 variant isoforms have been controversial in breast, colorectal and ovarian cancer (Mulder et al, 1994; Cannistra et al, 1995; Friedrichs et al, 1995; Kaufmann et al, 1995; Koretz et al, 1995; Uhl-Steidl et al, 1995).

In 44 patients with advanced ovarian cancer, tissue expression of CD44 splice variants, detected immunohistochemically by polyclonal antibody-recognizing variant exons v3–v10 of human CD44, was found to be associated with short disease-free and overall survival in univariate analysis (Uhl-Steidl et al, 1995). In an additional study, however, tissue expression of CD44s and its various alternatively spliced isoforms, as determined by monoclonal antibodies and reverse transcription polymerase chain reaction, failed to exhibit an association with survival in 31 ovarian cancer patients (Cannistra et al, 1995). These authors commonly detected CD44s and isoform v9, but not the isoforms v5 or v6, in ovarian cancer tissue (Cannistra et al, 1995). Accordingly, Sliutz et al (1995) demonstrated only traces of CD44v5 and v6 proteins in 2 out of 22 immunohistochemically investigated ovarian carcinoma samples. This is in agreement with our own observations based on immunohistochemical studies using the monoclonal antibodies VFF-8 and VFF-7, which recognize CD44 variant isoforms v5 and v6 respectively (data not shown).

Modest immunohistochemical detection alone does not necessarily exclude ovarian cancer cells as a source of soluble CD44 variant isoforms, as Yu and Toole (1996) recently demonstrated mRNA for CD44 isoforms that contained stop codons positioned so that translated proteins are truncated before the transmembrane domain and thus secreted directly as soluble molecules without transient membrane integration. However, the unconvincing immunohistochemical results in the detection of isoforms v5 and v6 in ovarian cancer tissue together with the findings of the current investigation, showing that CD44v5 serum content is lower in ovarian cancer patients than in healthy female control subjects and that splice variants v5 and v6 are present at significantly lower levels in ascitic fluid than in serum, indicate that ovarian cancer cells are not a relevant source of circulating CD44 isoforms v5 and v6.

As the present study reveals that soluble CD44v5 was inversely correlated with circulating immunostimulatory protein 90K, s-IL-2-R and serum neopterin, all of which belong to a group of molecules regarded as indicators of cellular immune-system activation (Marth et al, 1993; 1994; Zeimet et al, 1996), it cannot be ruled out that changes in systemic levels of soluble CD44v5 are due to tumour-induced alterations in the host's immune defence. This hypothesis is conceivable as expression of CD44 isoforms, including that of v5, in peripheral blood leucocytes was recently found to be inversely correlated to tumour progression in patients with haematological malignancies (Khaldoyanidi et al, 1996). The assumption of immunologically induced changes in systemic CD44v5 levels is further supported by the postulated pivotal role of cytokines in shedding soluble CD44 isoforms (Ristamäki et al, 1994).

To elucidate the involvement of the immune system in the regulation of circulating CD44 variant isoforms, we are currently investigating the constitutive and cytokine-modulated secretion of CD44 isoforms by immunocompetent cells under in vitro conditions. In addition, a prospective evaluation in a larger cohort is required to substantiate further the association of high circulating CD44v5 levels with favourable clinical course in ovarian cancer patients.

### ACKNOWLEDGEMENTS

This work was supported in part by a grant from the Fondation Lions Vaincre le Cancer Luxembourg. The authors thank Mrs Elisabeth Perkmann, Mrs Martina Fleischer and Mrs Hely Peterson for their excellent technical assistance.

### REFERENCES

Arch R, Wirth K, Hofmann M, Ponta H, Matzku S, Herrlich P and Zöller M (1992) Participation in normal immune responses of a metastasis-inducing splice variant of CD44. *Science* **257**: 682–685

Cannistra SA, Abu-Jawdeh G, Niloff J, Strobel T, Swanson L, Andersen J and Ottensmeier C (1995) CD44 variant expression is a common feature of epithelial ovarian cancer: Lack of association with standard prognostic factors. *J Clin Oncol* **13**: 1912–1921

Friedrichs K, Franke F, Lisboa BW, Kügler G, Gille I, Terpe HJ, Hölzel F, Maass H and Günthert U (1995) CD44 isoforms correlate with cellular differentiation but not with prognosis in human breast cancer. *Cancer Res* **55**: 5424–5433

Günthert U, Hofmann M, Rudy W, Reber S, Zoller M, Haussmann I, Matzku S, Wenzel A, Ponta H and Herrlich P (1991) A new variant of glycoprotein CD44 confers metastatic potential to rat carcinoma cells. *Cell* **65**: 13–24

Günthert U, Stauder R, Mayer B, Terpe HJ, Finke L and Friedrichs K (1995) Are CD44 variant isoforms involved in human tumour progression? *Cancer Surv* **24**: 19–42

Guo YJ, Liu G, Wang X, Jin D, Wu M, Ma J and Sy MS (1994) Potential use of soluble CD44 in serum as indicator of tumor burden and metastasis in patients with gastric or colon cancer. *Cancer Res* **54**: 422–426

© Cancer Research Campaign 1997

Heider KH, Dämmrich J, Skroch-Angel P, Müauller-Hermelink HK, Vollmers HP, Herrlich P and Ponta H (1993) Differential expression of CD44 splice variants in intestinal- and diffuse-type human gastric carcinomas and normal gastric mucosa. *Cancer Res* **53**: 4197–4203

Jalkannen SW, Bargatze RF, De Los Toyos J and Butcher EC (1987) Lymphocyte recognition of high endothelium: antibodies to distinct epitopes of an 85–95-kD glycoprotein antigen differentially inhibit lymphocyte binding to lymph node, mucosal or synovial endothelial cells. *J Cell Biol* **105**: 983–990

Kaufmann M, Heider KH, Sinn HP, Von Minckwitz G, Ponta H and Herrlich P (1995) CD44 variant exon epitopes in primary breast cancer and length of survival. *Lancet* **345**: 615–619

Khaldoyanidi S, Achtnich M, Hehlmann R and Zöller M (1996) Expression of CD44 variant isoforms in peripheral blood leukocytes in malignant lymphoma and leukemia: inverse correlation between expression and tumor progression. *Leukemia Res* **20**: 839–851

Koretz K, Möller P, Lehnert T, Hinz U, Otto HF and Herfarth C (1995) Effect of CD44v6 on survival in colorectal carcinoma. *Lancet* **345**: 327–328

Lein M, Weiß S, Jung K, Schnorr D, Loening S (1996) Soluble CD44 variants in serum of patients with prostate and other urological malignancies. *Prostate* **29**: 334–335

Marth C, Weger A, Müller-Holzner E, Zeimet AG, Moncayo-Naveda H, Daxenbichler G, Reibnegger G, Fuchs D, Wachter H and Dapunt O (1993) The prognostic value of nuclear roundness and neopterin in ovarian cancer. *Eur J Cancer* **29A**: 1863–1868

Marth C, Dreps A, Natoli C, Zeimet AG, Lang T, Widschwendter M, Daxenbichler G, Ullrich A and Iacobelli S (1994) Effects of type-I and II interferons on 90K antigen expression in ovarian carcinoma cells. *Int J Cancer* **59**: 808–813

Mayer B, Jauch KW, Günthert U, Figdor CG, Schildberg FW, Funke I and Johnson JP (1993) De-novo expression of CD44 and survival in gastric cancer. *Lancet* **342**: 1019–1022

Miyake K, Underhill CB, Lesley J and Kincade PW (1990) Hyaluronate can function as a cell adhesion molecule and CD44 participates in hyaluronate recognition. *J Exp Med* **172**: 69–75

Mulder JWR, Kruyt PM, Sewnath M, Oosting J, Seldenrijk CA, Weidema WF, Offerhaus GJA and Pals ST (1994) Colorectal cancer prognosis and expression of exon-v6-containing CD44 proteins. *Lancet* **344**: 1470–1472

Ristamäki BR, Joensuu H, Salmi M and Jalkanen S (1994) Serum CD44 in malignant lymphoma: an association with treatment response. *Blood* **84**: 238–243

Salles G, Zain M, Jiang WM, Boussiotis VA and Shipp MA (1993) Alternatively spliced CD44 transcripts in diffuse large cell lymphomas: characterization and comparison with normal activated B cells and epithelial malignancies. *Blood* **82**: 3539–3547

Seiter S, Arch R, Reber S, Komitowski D, Hofmann M, Ponta H, Herrlich P, Matzku S and Zoller M (1993) Prevention of tumour metastasis formation by anti-variant CD44. *J Exp Med* **177**: 443–455

Sliutz G, Tempfer C, Winkler S, Kohlberger P, Reinthaller A and Kainz C (1995) Immunohistochemical and serological evaluation of CD44 splice variants in human ovarian cancer. *Br J Cancer* **72**: 1494–1497

Stauder R, Eisterer W, Thaler J and Günthert U (1995) CD44 variant isoforms in non-Hodgkin's lymphoma: A new independent prognostic factor. *Blood* **85**: 2885–2899

Terpe HJ, Stark H, Prehm P and Günthert U (1994) CD44 variant isoforms are preferentially expressed in basal epithelia of non-malignant human fetal and adult tissues. *Histochemistry* **101**: 79–89

Uhl-Steidl M, Müller-Holzner E, Zeimet AG, Adolf GR, Daxenbichler G, Marth C and Dapunt O (1995) Prognostic value of CD44 splice variant expression in ovarian cancer. *Oncology* **52**: 400–406

Yu Q and Toole BP (1996) A new alternatively spliced exon between v9 and v10 provides a molecular basis for synthesis of soluble CD44. *J Biol Chem* **271**: 20603–20607

Zeimet AG, Natoli C, Herold M, Fuchs D, Windbichler G, Daxenbichler G, Iacobelli S, Dapunt O and Marth C (1996) Circulating immunostimulatory protein 90K and soluble interleukin-2-receptor in human ovarian cancer. *Int J Cancer* **68**: 34–38

© Cancer Research Campaign 1997